# Defective Neural Stem and Progenitor Cell Proliferation in Neurodevelopmental Disorders

**DOI:** 10.3390/jdb13040040

**Published:** 2025-11-07

**Authors:** Aki Shigenaka, Eri Nitta, Tadashi Nakagawa, Makiko Nakagawa, Toru Hosoi

**Affiliations:** 1 Department of Clinical Pharmacology, Faculty of Pharmaceutical Sciences, Sanyo-Onoda City University, Sanyo-Onoda 756-0884, Japan; p120050@ed.socu.ac.jp (A.S.); hosoi@rs.socu.ac.jp (T.H.); 2 Division of Cell Proliferation, United Centers for Advanced Research and Translational Medicine, Graduate School of Medicine, Tohoku University, Sendai 980-8575, Japan; 3 Institute of Gene Research, Science Research Center, Yamaguchi University, Ube 755-8505, Japan; mnakagaw@yamaguchi-u.ac.jp; 4 Advanced Technology Institute, Life Science Division, Yamaguchi University, Yamaguchi 755-8611, Japan

**Keywords:** neural stem and progenitor cells, neurodevelopmental disorders, cell cycle regulation, signal transduction, chromatin remodeling, microcephaly and macrocephaly, environmental teratogens

## Abstract

Neurodevelopmental disorders (NDDs), including autism spectrum disorder, intellectual disability, and attention deficit hyperactivity disorder, are increasingly recognized as disorders of early brain construction arising from defects in neural stem and progenitor cell (NSPC) proliferation. NSPCs are responsible for generating the diverse neuronal and glial lineages that establish cortical architecture and neural circuitry; thus, their expansion must be tightly coordinated by intrinsic cell cycle regulators and extrinsic niche-derived cues. Disruption of these mechanisms—through genetic mutations, epigenetic dysregulation, or environmental insults—can perturb the balance between NSPC self-renewal and differentiation, resulting in aberrant brain size and connectivity. Recent advances using animal models and human pluripotent stem cell-derived brain organoids have identified key signaling pathways, including Notch, Wnt, SHH, and PI3K–mTOR, as central hubs integrating proliferative cues, while transcriptional and chromatin regulators such as PAX6, CHD8, SETD5, and ANKRD11 govern gene expression essential for proper NSPC cycling. Furthermore, prenatal exposure to teratogens such as Zika virus infection, valproic acid, or metabolic stress in phenylketonuria can recapitulate proliferation defects and microcephaly, underscoring the vulnerability of NSPCs to environmental perturbation. This review summarizes emerging insights into the molecular and cellular mechanisms by which defective NSPC proliferation contributes to NDD pathogenesis, highlighting convergence among genetic and environmental factors on cell cycle control. A deeper understanding of these pathways may uncover shared therapeutic targets to restore neurodevelopmental trajectories and mitigate disease burden.

## 1. Introduction

Neurodevelopmental disorders (NDDs) encompass a broad spectrum of conditions including autism spectrum disorder (ASD), intellectual disability (ID), and attention deficit hyperactivity disorder (ADHD) [1]. These disorders affect approximately 1 in 20 children globally [2] and pose significant lifelong challenges for affected individuals and their families [3,4]. Despite their clinical heterogeneity, these disorders are believed to share a common underlying feature: the disruption of early neurodevelopmental processes critical for establishing proper brain architecture and function [5,6].

**Figure 1:**
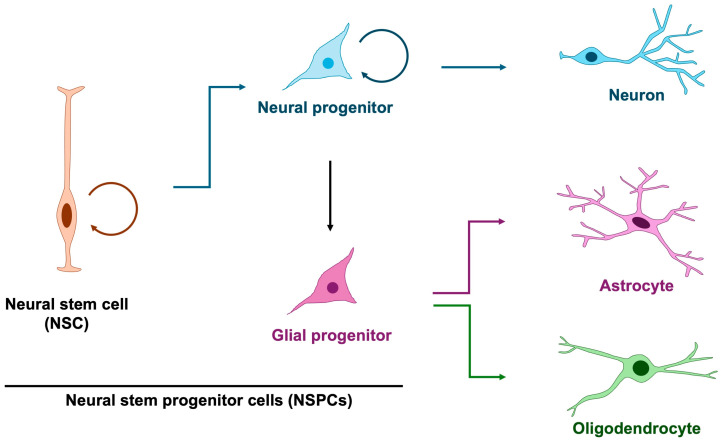
Differentiation trajectory of neuron, astrocyte and oligodendrocyte. In principle, glial cells arise from glial progenitors; however, studies on neural stem and progenitor cells have demonstrated that neural progenitors themselves exhibit glial-like properties and can also function as glial progenitors. Three-quarter circular arrows denote self-renewal. Blue arrows indicate neurogenesis, purple arrows represent astrogenesis, and green arrows denote oligodendrogenesis.

Among the earliest processes in brain formation is the proliferation of embryonic cortical neural stem and progenitor cells (NSPCs), which give rise to the neurons and glial cells that populate the central nervous system [5,7,8]. In principle, neural stem cells generate neural progenitors that predominantly give rise to neurons, whereas glial progenitors are thought to exclusively produce astrocytes and oligodendrocytes (Figure 1). However, it has revealed that neural progenitor cells exhibit glial characteristics and are capable of producing not only neurons but also astrocytes and oligodendrocytes [9], thereby blurring the distinction between dedicated glial progenitors and multipotent neural progenitors. Notably, neurogenesis precedes gliogenesis, commencing at approximately embryonic day 10 in mice (gestational week 10 in humans) and shifting toward gliogenesis around embryonic day 16 in mice (gestational week 16 in humans) [10,11,12]. This temporal progression indicates a dynamic change in the developmental potential of neural progenitor cells. Although mounting evidence implicates glial abnormalities in NDDs [13,14], the specific contribution of aberrant gliogenesis remains less well defined. Accordingly, in this review, we highlight defective NSPC proliferation and its relevance to the pathogenesis of NDDs.

**Figure 2:**
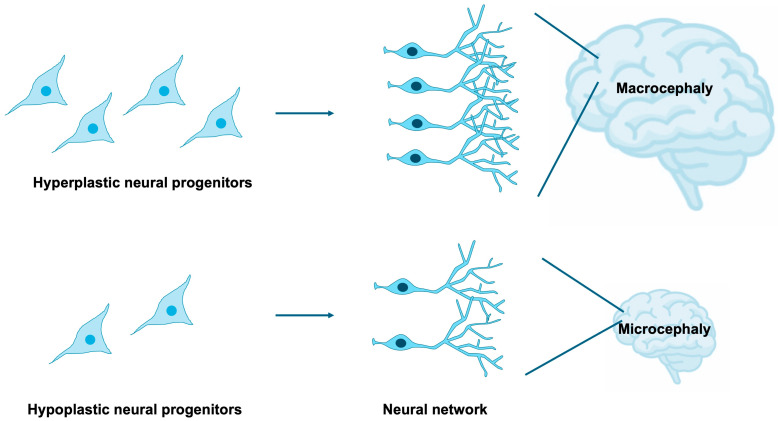
Impact of NSPC proliferation on brain development. The extent of NSPC expansion through proliferative activity critically influences overall brain size. Macrocephaly and microcephaly are clinically defined as head circumferences exceeding or falling below two standard deviations from the population mean, respectively. Excessive proliferation of neural progenitors gives rise to macrocephaly, whereas insufficient proliferation results in microcephaly.

NSPCs are highly sensitive to both intrinsic and extrinsic cues that govern their proliferation, survival, and differentiation [15,16,17,18]. Tight regulation of NSPC expansion and lineage specification is indispensable for the proper establishment of brain size, cortical lamination, and neural circuitry [19,20,21,22]. Disruptions in these processes can yield profound neuroanatomical and functional abnormalities, exemplified by microcephaly and macrocephaly [23,24,25], as well as aberrant synaptic organization frequently observed in neurodevelopmental disorders [26] (Figure 2). It is estimated that 15–79% of individuals with NDDs exhibit comorbid microcephaly [27,28], while 54–67% of patients with microcephaly present with NDDs [27,29], indicating that microcephaly constitutes a frequent and clinically significant comorbidity of NDDs. Furthermore, approximately 16–22% of individuals with NDDs display comorbid macrocephaly [30,31], and 17% of patients with macrocephaly are diagnosed with NDDs [32], suggesting that macrocephaly also represents a relatively common and noteworthy comorbid condition, albeit with a weaker correlation compared to microcephaly.

Leveraging animal models and pluripotent stem cell-derived NSPCs, recent studies have implicated a diverse spectrum of genetic factors—many of which are mutated in patients with NDDs—in the regulation of NSPC proliferation [33,34]. In parallel, NDD-associated environmental insults during gestation have also been shown to perturb NSPC proliferation [35,36,37,38]. Collectively, these findings underscore defective NSPC proliferation as a critical early pathogenic event in NDDs, emphasizing the necessity of elucidating this process to inform the development of targeted therapeutic strategies. In this review, we summarize current knowledge of the molecular mechanisms underlying aberrant NSPC proliferation, with particular focus on the regulation of the cell division cycle, a fundamental determinant of NSPC expansion.

## 2. Neural Stem and Progenitor Cells in Normal Brain Development

**Figure 3:**
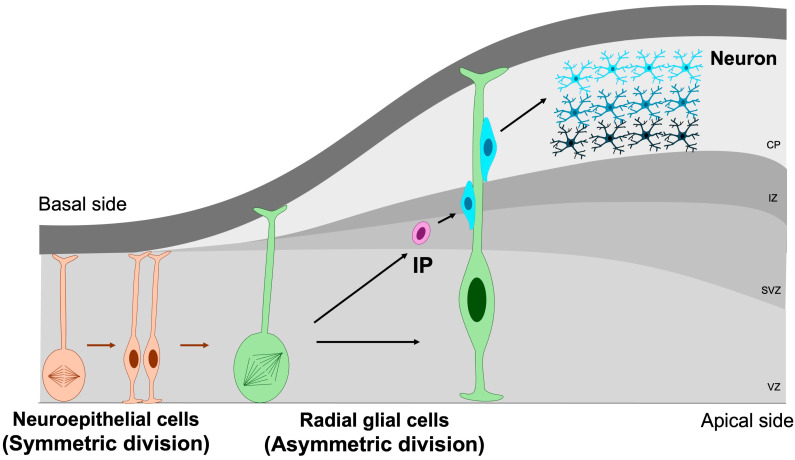
Symmetric and asymmetric division of NSPCs. Symmetric division is essential for maintaining the NSPC pool through self-renewal, whereas asymmetric division drives the generation of differentiated neuronal lineages. IP, intermediate progenitor; VZ, ventricular zone; SVZ, subventricular zone; IZ, intermediate zone; CP, cortical plate. Orange cells represent neuroepithelial cells, green cells denote radial glial cells, purple cells correspond to intermediate progenitors, and blue cells indicate mature neurons. Orange arrows indicate the differentiation of neuroepithelial cells, whereas green arrows represent the differentiation of radial glial cells.

The earliest NSPCs in the developing brain are neuroepithelial cells, a population of neural stem cells that initially undergo symmetric divisions to expand the progenitor pool during early neurogenesis [7]. As the neural tube matures, these neuroepithelial cells progressively transform into radial glial cells—highly polarized progenitors that extend across the entire thickness of the embryonic cortex. Radial glia generate both neurons and intermediate progenitors through symmetric and asymmetric divisions [39]. Intermediate progenitors (IPs), residing predominantly within the subventricular zone, possess more restricted developmental potential and primarily give rise to neurons via neurogenic divisions [40,41] (Figure 3).

NSPC proliferation is orchestrated by a complex interplay of intrinsic and extrinsic mechanisms. Intrinsic regulation encompasses mitotic spindle orientation, chromatin remodeling, and the expression of lineage-determining transcription factors [42,43]. For instance, the balance between symmetric and asymmetric divisions, essential for self-renewal and differentiation, respectively, is critically shaped by the orientation of the mitotic spindle and the polarized distribution of cell fate determinants [20,44]. Extrinsic signals from the neural stem cell niche—including Notch, Wnt, Sonic Hedgehog (SHH), fibroblast growth factor (FGF), and insulin-like growth factor-I (IGF-I)—further modulate NSPC behavior [45,46,47,48,49,50]. In addition, interactions with the extracellular matrix and local metabolic states play pivotal roles in regulating proliferative capacity and lineage specification [51,52].

## 3. Cell Cycle Regulation in NSPC Proliferation

**Figure 4:**
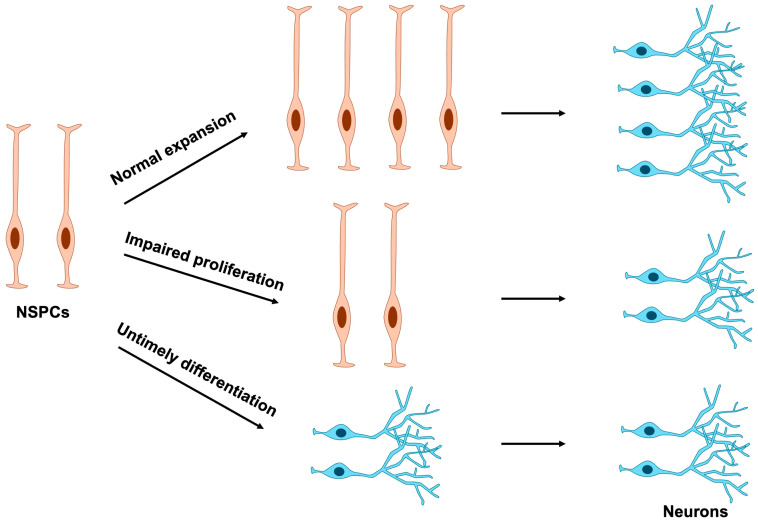
Effects of NSPC proliferation and differentiation on neuronal number. Normal expansion of NSPCs ensures the proper production of mature neurons. In contrast, impaired proliferation or premature differentiation of NSPCs results in a marked reduction in neuronal numbers. NSPCs, neural stem and progenitor cells.

Because terminally differentiated cells have lost their proliferative capacity, disruptions in NSPC proliferation can arise through two principal mechanisms: direct perturbation of cell division or indirect perturbation mediated by aberrant differentiation timing (Figure 4). Consequently, abnormal NSPC proliferation often stems from mutations in genes that compromise the regulation of either cell cycle progression or differentiation.

**Figure 5:**
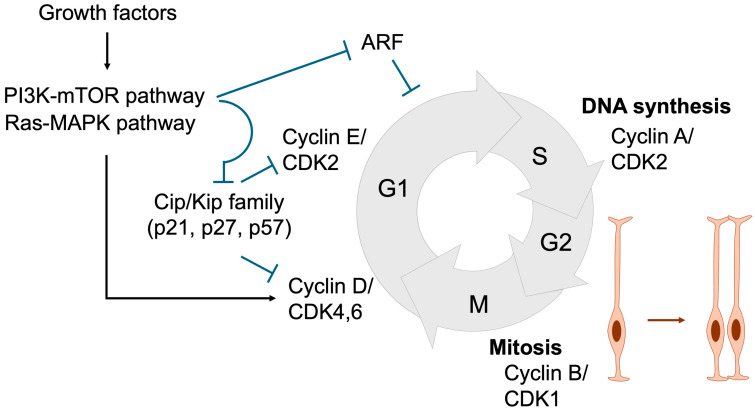
Cell cycle regulation essential for NSPC expansion. During the G1 phase, Cyclin D and Cyclin E are sequentially expressed, resulting in the activation of CDK4/6 and CDK2. Growth factors promote G1 phase progression by inducing Cyclin D expression and suppressing Cip/Kip family CDK inhibitors (p21, p27, and p57) through the Ras-MAPK and PI3K-mTOR signaling pathways, respectively. ARF also acts as a negative regulator of G1 progression. In the S phase, Cyclin A expression activates CDK2, thereby initiating DNA replication. During the G2 phase, Cyclin B accumulation leads to CDK1 activation, which governs entry into and progression through the M phase, where cell division occurs. Black arrows denote activation, whereas blue T-shaped arrows represent inhibition. Orange arrow represents the proliferation of neural stem cells.

Proper and timely cell division is tightly regulated through coordinated control of cell cycle progression. During the G1 phase, diploid cells prepare for DNA synthesis, which occurs in the S phase. The resulting tetraploid cells then enter the G2 phase, where they prepare for mitosis, ultimately producing two daughter cells in the M phase [43,53,54] (Figure 5). In response to growth factors described in Section 2, signaling cascades such as the MAPK and PI3K/mTOR pathways are activated, leading to the induction of Cyclin D. Cyclin D subsequently activates CDK4/6, which is required for entry into S phase and DNA replication. Cyclin D–CDK4/6 activity further induces Cyclin E, which activates CDK2 to drive G1 phase progression. Thereafter, the Cyclin A–CDK2 complex plays a pivotal role in initiating DNA replication by activating the replication machinery, while also coordinating the timely activation of the Cyclin B–CDK1 complex, which governs progression into mitosis.

The duration of the cell cycle is a critical determinant of NSPC proliferation and differentiation. As NSPCs progress toward neuronal differentiation, the overall cell cycle lengthens, predominantly due to an extended G1 phase. This prolonged G1 phase is thought to be essential, as it provides sufficient time for the accumulation and activity of fate-determining factors that promote differentiation [43,55]. Consistently, experimental elongation of the G1 phase via CDK inhibitors drives NSPC differentiation [56]. Conversely, artificial shortening of the G1 phase through overexpression of Cyclin D or Cyclin E delays differentiation and maintains NSPCs in a proliferative, undifferentiated state [57,58]. These findings indicate that inappropriate inhibition of cell cycle progression can prematurely trigger differentiation and deplete the NSPC pool, thereby contributing to the pathogenesis of NDDs.

## 4. Impairment of NSPC Proliferation in Neurodevelopmental Disorders

Abnormal cell cycle progression is largely driven by dysregulated expression of key cell cycle regulators, often as a consequence of disrupted signaling pathways [59,60], aberrant activity of transcription factors, and altered chromatin remodeling [61,62,63]. These perturbations collectively impair NSPC proliferation and compromise normal brain development.

### 4.1. Genetic Factors

#### 4.1.1. Signaling Pathways

**Figure 6:**
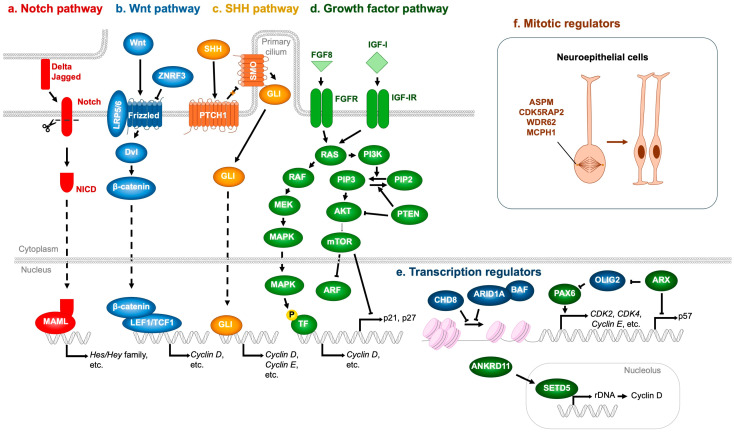
Molecular regulators whose mutations contribute to impaired NSPC proliferation and NDDs. (**a**) Notch pathway. Ligands expressed on adjacent cells (Delta, Jagged) activate the Notch receptor, generating the Notch intracellular domain (NICD), which associates with MAML to induce the transcription of *Hes* and *Hey* family genes. (**b**) Wnt pathway. Wnt ligands bind to the LRP5/6–Frizzled receptor complex, recruiting Dvl and stabilizing β-catenin, which translocates into the nucleus and binds to LEF1/TCF1 transcription factors to promote target gene expression, including *Cyclin D*. ZNRF3 acts as a negative regulator by ubiquitinating and degrading Frizzled receptors. (**c**) SHH pathway. Binding of SHH to PTCH1 relieves its inhibition of SMO, enabling GLI transcription factors to enter the nucleus and activate downstream genes such as *Cyclin D* and *Cyclin E*. (**d**) Growth factor pathway. Representative ligands such as FGF8 and IGF-I activate their respective receptors (FGFR and IGF-IR), initiating downstream cascades. The RAS–MAPK pathway (RAS → RAF → MEK → MAPK) promotes transcription of *Cyclin D*, whereas the PI3K–mTOR pathway (PI3K → AKT → mTOR) suppresses cell cycle inhibitors including ARF, p21, and p27. (**e**) Transcription regulators. The CHD8 and ARID1A-containing BAF complex represses, whereas PAX6 enhances, the transcription of cell cycle activators such as *CDK2*, *CDK4*, and *Cyclin E*. ARX indirectly upregulates PAX6 expression through *OLIG2* repression and concurrently inhibits the expression of the cell cycle inhibitor *p57*. Furthermore, ANKRD11 enhances rDNA transcription and subsequently induces *Cyclin D* expression via SETD5 upregulation. (**f**) Mitotic regulators. ASPM, CDK5RAP2, WDR62, and MCPH1 are essential for proper mitotic spindle assembly, thereby ensuring accurate neuroepithelial cell division. T-shaped arrows represent inhibition, whereas dotted arrows denote indirect activation. Orange arrow represents the proliferation of neural stem cells.

Notch signaling is a master regulator of NSPC proliferation. Upon ligand activation (Jagged, Delta), Notch receptors undergo proteolytic cleavage to release the Notch intracellular domain (NICD), which associates with RBPJ and MAML to drive transcription of target genes, notably the *Hes* and *Hey* families [64] (Figure 6a). Hes and Hey proteins repress proneural genes, thereby maintaining NSPCs in an undifferentiated state and preventing premature neuronal differentiation [65]. Mutations in *NOTCH2NL* (encoding a Notch receptor) and *DLL1* (encoding a Delta ligand) have been identified in NDD patients [66,67]. Importantly, *NOTCH2NL* deletions are associated with microcephaly, whereas duplications result in macrocephaly [66]. Similarly, *DLL1* mutations have been linked to microcephaly [67], underscoring the essential role of Notch signaling in NSPC expansion.

The Wnt pathway is also pivotal in regulating NSPC proliferation. In the absence of Wnt ligands, β-catenin is continuously targeted for degradation by the destruction complex [68]. Wnt binding to Frizzled and LRP5/6 recruits Dishevelled (Dvl), sequestering the destruction complex at the membrane and preventing β-catenin degradation. Stabilized β-catenin then accumulates, translocates to the nucleus, and partners with TCF/LEF transcription factors to activate target genes such as *Cyclin D*, which promote NSPC proliferation [69,70] (Figure 6b). Pathogenic missense variants in *ZNRF3*, a negative regulator of Wnt signaling that ubiquitinates and inactivates Frizzled, have been identified in NDD patients [71]. Variants clustered in the RING domain impair Frizzled ubiquitination, leading to excessive Wnt activity, aberrant NSPC proliferation, and macrocephaly. Conversely, microcephaly-associated *ZNRF3* variants localize to the RSPO-binding domain, conferring resistance to RSPO inhibition and resulting in hyperactive Frizzled degradation, diminished Wnt signaling, reduced NSPC proliferation, and microcephaly [71,72].

The SHH pathway likewise regulates NSPC proliferation. SHH binding to the PTCH1 receptor relieves its inhibition of Smoothened (SMO), thereby enabling SMO activation [73]. Activated SMO promotes nuclear translocation of GLI transcription factors, which induce expression of proliferation-associated genes, including *Cyclin D* and *Cyclin E* [73] (Figure 6c). Consistently, mutations in *SHH* or *PTCH1* have been identified in patients with microcephalic NDDs [74,75].

FGF and IGF-I also critically regulate NSPC proliferation. These ligands bind to their receptors (FGFR and IGF-IR, respectively), activating shared downstream pathways—RAS-MAPK (RAS → RAF → MEK → MAPK) and PI3K-mTOR (PI3K → AKT → mTOR)—that are most extensively characterized [76,77,78]. The MAPK cascade phosphorylates transcription factors that upregulate cell cycle drivers such as *Cyclin D* [79,80], while mTOR promotes progression by degrading ARF [81], and repressing CDK inhibitors *p21* and *p27* [82] (Figure 6d). PI3K-mediated phosphorylation of PIP2 to PIP3 activates AKT, whereas PTEN antagonizes this pathway by dephosphorylating PIP3 back to PIP2, thereby acting as a brake on NSPC proliferation [83]. The pathological relevance of these signaling cascades to NDDs is underscored by numerous genetic findings: loss-of-function mutations in *FGFR1* [84], *FGFR3* [85], *FGF8* [86,87], *IGF-1* [88,89,90], *IGF-IR* [91,92], and *AKT3* [93] have been linked to microcephalic NDDs. Conversely, loss-of-function mutations in *PTEN* [94,95,96,97], stabilizing mutations in *Cyclin D2* [98], and activating mutations in *PI3K* [97,99,100], *AKT3* [93,99,100], and *mTOR* [97,99,101] are consistently associated with macrocephalic NDDs.

#### 4.1.2. Transcription Factors

In addition to the signaling pathways described above, several transcriptional regulators critically modulate NSPC proliferation. Loss of function of the transcription factor *ARX* has been associated with NDDs accompanied by microcephaly [102]. *ARX* deficiency leads to upregulation of *p57*, a Cip/Kip family CDK inhibitor (Figure 6e), which is normally repressed by ARX. This results in premature cell cycle exit and depletion of progenitor pools [103]. Moreover, ARX loss elevates expression of the transcription factor *OLIG2* [104], which in turn reduces neurogenesis and contributes to overall brain size reduction [105]. Mechanistically, OLIG2 overexpression downregulates *PAX6*, a key transcription factor governing both proliferation and differentiation of cortical progenitors [104,105].

PAX6 positively regulates several cell cycle-promoting genes, including *CDK2*, *CDK4*, and *Cyclin E*, thereby enhancing NSPC proliferation during embryonic brain development [106] (Figure 6e). Consistently, compound heterozygous *PAX6* mutations are associated with microcephaly in human patients [107,108]. Interestingly, PAX6 also functions as a repressor of NSPC cell cycle progression, as evidenced by studies in mice carrying *Pax6* mutations [109] or conditional *Pax6* deletions in developing neurons [110]. This inhibitory effect is likely mediated by suppression of CDK6 expression. Supporting evidence includes PAX6 binding to regulatory regions surrounding the *Cdk6* locus [110], rapid upregulation of *Cdk6* following acute Pax6 loss [111], and reduced NSPC proliferation upon CDK6 inhibition or gene knockout [110,112]. Consistently, *CDK6* mutations have been linked to microcephaly [113].

Notably, PAX6 simultaneously promotes neuronal differentiation by activating neurogenic gene expression, suggesting a dual role in balancing NSPC proliferation and neurogenesis [106]. Indeed, experimental evidence from *Pax6*-mutant mice demonstrates that Pax6 loss reduces NSPC proliferation, whereas Pax6 overexpression accelerates neuronal differentiation at the expense of progenitor expansion [106]. Thus, both upregulation and downregulation of *Pax6* ultimately converge on a common outcome—microcephaly. This sensitivity is partly managed by its antagonistic interactions within a network of neurogenic transcription factors such as Neurog2, Ascl1, Hes1 [106]. These findings suggest that the seemingly paradoxical roles of PAX6—in both promoting and restraining NSPC proliferation—are governed by context-dependent mechanisms, including gene dosage effects and interactions with other transcriptional regulators.

#### 4.1.3. Chromatin Remodelers

Mutations in *CHD8*, a gene encoding a chromatin remodeler, have been strongly associated with macrocephaly, suggesting that CHD8 acts as a transcriptional brake on NSPC proliferation. Consistently, haploinsufficiency of CHD8—commonly observed in NDD patients—shortens the cell cycle and enhances NSPC proliferation [114]. This phenotype has been attributed to the upregulation of *Cyclin E*, a key driver of S-phase entry [114] (Figure 6e). Similarly, germline *Chd8* haploinsufficiency in mice accelerates NSPC proliferation, accompanied by dysregulated expression of cell cycle-associated genes [115]. Although these findings support a role for CHD8 as a negative regulator of cell proliferation, other studies have reported that CHD8 promotes NSPC proliferation at both cellular and organismal levels [116,117]. These discrepancies may reflect methodological differences, as studies reporting pro-proliferative roles primarily employed mRNA knockdown rather than genetic knockout models, suggesting that the degree of CHD8 reduction critically influences NSPC outcomes.

Gene dosage effects on chromatin remodeling also extend to components of the BAF (SWI/SNF) complex. Duplication of *ARID1A* (also known as *BAF250A*), a core subunit of the BAF complex, has been associated with microcephaly [118]. Conversely, haploinsufficiency of *ARID1B* (also known as *BAF250B*), a mutually exclusive paralog of *ARID1A*, similarly results in microcephaly [119,120,121], implying antagonistic roles in NSPC proliferation—with ARID1A-BAF functioning as a repressor and ARID1B-BAF as an activator. Supporting this notion, conditional deletion of *Arid1a* in murine NSPCs enhances proliferation and upregulates cell cycle-related genes [122] (Figure 6e). By contrast, the role of ARID1B in NSPC proliferation remains largely unexplored. Nonetheless, *Arid1b*-deficient murine embryonic stem cells, as well as patient-derived fibroblasts with *ARID1B* haploinsufficiency, exhibit reduced proliferation accompanied by downregulation of CDC20, a key positive regulator of cell cycle progression [123,124]. Whether NSPC proliferation is similarly attenuated by ARID1B reduction remains an open question warranting further investigation.

Another gene whose mutation disrupts NSPC proliferation through transcriptional dysregulation is SETD5, an epigenetic regulator that modulates rDNA transcription and translation of key cell cycle proteins such as *Cyclin D* [125] (Figure 6e). Haploinsufficiency of *SETD5* leads to reduced rRNA synthesis and impaired NSPC proliferation [125]. In humans, heterozygous loss-of-function mutations in *SETD5* cause a neurodevelopmental disorder termed IDD23 (also known as MRD23) [126]. Intriguingly, pathogenic *SETD5* variants have also been identified in patients presenting with KBG-like phenotypes (KBG designation originates from the initials of the surnames of the three families originally described with the condition [127])—syndromes that phenotypically overlap with KBG syndrome [128,129].

Haploinsufficiency of another epigenetic regulator, *ANKRD11*, is recognized as the primary genetic cause of KBG syndrome, suggesting a functional interplay between ANKRD11 and SETD5 [126]. Supporting this notion, recent studies have shown that ANKRD11 positively regulates *SETD5* expression, thereby sustaining translational capacity and neuronal proliferation—processes essential for mitigating NDD pathogenesis [130] (Figure 6e). Notably, both IDD23 and KBG syndrome patients frequently exhibit microcephaly, underscoring the crucial role of SETD5- and ANKRD11-mediated control of NSPC proliferation in maintaining proper brain development [131,132,133].

#### 4.1.4. Centrosomal and Spindle Proteins

Key regulators that directly control NSPC proliferation also include centrosomal and spindle apparatus proteins such as ASPM, CDK5RAP2, WDR62, and MCPH1, mutations in which are causatively linked to microcephaly in humans [134,135]. Defects in these genes disrupt mitotic spindle orientation and impair symmetric cell divisions, resulting in premature depletion of the progenitor pool and consequent reduction in cortical size [134,135] (Figure 6f).

### 4.2. Environmental Factors

**Figure 7:**
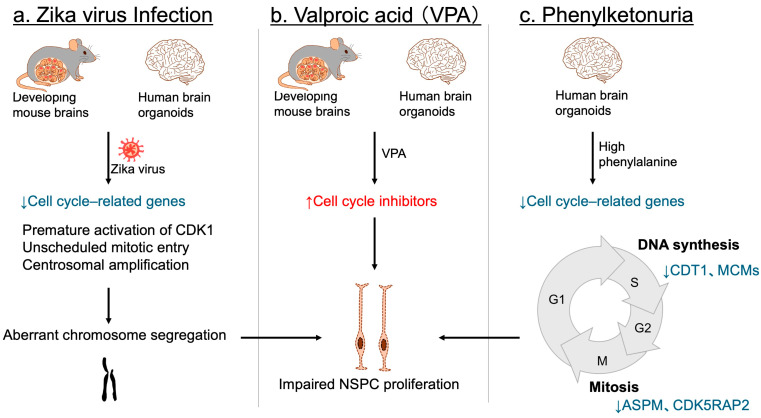
Environmental factors leading to impaired NSPC proliferation and NDDs. (**a**) In both mouse embryonic brains and human cerebral organoids, Zika virus infection disrupts the expression of key cell cycle regulators. It also triggers premature CDK1 activation, unscheduled mitotic entry, and centrosomal amplification, resulting in aberrant chromosome segregation, and subsequent impairment of NSPC proliferation. (**b**) Valproic acid (VPA) exposure. In both mouse models and human brain organoids, exposure to VPA upregulates cell cycle inhibitors, thereby suppressing NSPC proliferation. (**c**) Phenylketonuria. In human brain organoids, elevated phenylalanine concentrations downregulate genes essential for cell cycle progression—such as *CDT1* and *MCM* family members involved in the S phase, and *ASPM* and *CDK5RAP2* associated with the M phase—ultimately leading to defective NSPC proliferation. Blue arrows and letters denote downregulation, whereas red arrows and letters indicate upregulation.

Environmental factors can also profoundly impact NSPC proliferation. Maternal infection with the Zika virus has been firmly established as a cause of congenital microcephaly and NDDs in offspring [136,137,138,139]. Consistent with these observations, Zika virus infection disrupts NSPC proliferation in developing mouse brains [140,141], as well as in human three-dimensional brain organoids [142,143]. Although numerous cell cycle-related genes are downregulated [144,145], accumulating evidence indicates that premature activation of CDK1 and unscheduled mitotic entry, together with centrosomal amplification, lead to aberrant chromosome segregation, resulting in NSPC proliferation defects and cell death [144,145,146,147] (Figure 7a). This cascade is considered a primary mechanism underlying the proliferation defects observed in Zika virus-infected NSPCs.

In utero drug exposure also contributes to NDDs associated with microcephaly. Valproic acid (VPA), a widely used antiepileptic and mood stabilizer, has been shown to increase the risk of NDDs with microcephaly [148,149]. Experimental studies in mice have demonstrated that embryonic exposure to VPA induces microcephaly and behavioral phenotypes resembling NDDs [150,151,152], ruling out the possibility that maternal psychiatric conditions alone account for the observed abnormalities. Mechanistically, VPA exposure impairs NSPC proliferation in mice through upregulation of several cell cycle inhibitors [152] (Figure 7b). In human brain organoids, ARF—but not other CDK inhibitors—was significantly induced following VPA treatment, implicating ARF as a key mediator of NSPC proliferation defects [152] (Figure 7b).

Another environmental and metabolic risk factor for microcephalic NDDs is phenylketonuria (PKU), arising from either maternal or inborn deficiency [153,154]. Most PKU cases result from pathogenic loss-of-function variants in *PAH*, encoding phenylalanine hydroxylase, the enzyme responsible for converting phenylalanine to tyrosine. This defect leads to elevated phenylalanine levels in both blood and the brain [153,154]. Studies using human iPSC-derived brain organoids have demonstrated that high phenylalanine concentrations selectively impair NSPC populations, while mature neurons and glia remain largely unaffected, highlighting the heightened vulnerability of NSPCs to metabolic stress [155]. Transcriptomic profiling further demonstrated a broad downregulation of genes essential for cell cycle progression, including *CDT1* and *MCMs* involved in the S phase, as well as *ASPM* and *CDK5RAP2* associated with the M phase [155] (Figure 7c). These findings suggest that impaired NSPC proliferation, driven by defective cell cycle regulation, directly contributes to microcephalic NDD in PKU.

## 5. Future Directions and Therapeutic Perspectives

The growing body of evidence implicating defective NSPC proliferation in the etiology of NDDs highlights the urgent need to translate mechanistic insights into therapeutic strategies. Future research should aim to delineate how distinct genetic mutations, epigenetic dysregulation, and environmental exposures converge on shared cell cycle and signaling pathways in NSPCs. Integration of single-cell transcriptomics, proteomics, and epigenomics with brain organoid models and in vivo systems will enable the identification of temporal and lineage-specific vulnerabilities that underlie aberrant proliferation. Such high-resolution approaches may reveal common “molecular bottlenecks” in NSPC regulation, offering tractable targets for intervention.

Cell cycle regulation is not unique to NSPCs. Notably, epidemiological studies indicate that individuals with NDDs exhibit an elevated susceptibility to various forms of cancer [156,157,158,159], and approximately one-third of NDD risk genes overlap with cancer driver genes [160]. These finding suggest that NDD-associated genes may influence not only neural but also other stem cell populations. Elucidating the roles of NDD-affiliated genes in diverse stem cell types will provide deeper insight into both their shared and cell type-specific functions, as well as the selective vulnerability of distinct stem cell lineages.

From a therapeutic standpoint, several avenues hold promise. Pharmacological modulation of core signaling cascades—such as Notch, Wnt, SHH, and PI3K–mTOR—could restore balanced NSPC proliferation, provided that interventions are carefully titrated to avoid exacerbating either hypo- or hyperplastic states. Small-molecule inhibitors of CDKs or pathway-specific agonists/antagonists represent potential candidates, some of which are already under clinical investigation for oncology and may be repurposed for NDDs. Epigenetic regulators such as CHD8, SETD5, and ANKRD11, whose dysfunction directly impairs NSPC proliferation, are also attractive therapeutic nodes, particularly as pharmacological modulators of chromatin structure and transcriptional machinery continue to expand. Nevertheless, because most NSPC proliferation is completed by birth, direct postnatal pharmacological intervention remains challenging. Therefore, prenatal genetic diagnosis of NDD-related mutations may be crucial for the effective application of NSPC-targeted therapeutic strategies.

In addition to proliferation, other facets of NSPC biology are crucial for proper brain development, and their dysregulation has been implicated in NDDs. For instance, aberrant migration of NSPCs caused by mutations in more than 30 genes, including several tubulin and actin genes, leads to cortical malformations and mislamination, resulting in a smooth brain surface (lissencephaly) associated with NDDs [161]. Likewise, disrupted differentiation balance between neuronal and glial lineages contributes to abnormal synaptogenesis, cortical circuit assembly, and network connectivity [162,163]. Collectively, these findings indicate that perturbations at every stage of NSPC biology—proliferation, differentiation, and migration—contribute to NDD pathogenesis. As discussed in this review, these processes are closely linked to cell cycle progression. Elucidating their molecular interconnections is crucial for understanding the intricate mechanisms underlying NDD pathogenesis associated with NSPC dysfunction. We hope this review will inspire researchers in the field to address these critical knowledge gaps.

Ultimately, future efforts will benefit from interdisciplinary approaches that link developmental neurobiology, stem cell biology, and translational neuroscience. By bridging the gap between mechanistic understanding and therapeutic application, these studies have the potential not only to uncover predictive biomarkers of disease severity but also to lay the groundwork for preventive and restorative interventions in NDDs. The challenge ahead is to transform knowledge of NSPC biology into tangible therapies that can improve neurodevelopmental trajectories and quality of life for affected individuals.

## Data Availability

No new data were created or analyzed in this study.

## Abbreviations

The following abbreviations are used in this manuscript:

ADHD	Attention deficit hyperactivity disorder
ANKRD11	Ankyrin repeat domain containing 11
ARID	AT-rich interaction domain
ASPM	Assembly factor for spindle microtubules
BAF	Barrier-to autointegration factor
CDK	Cyclin dependent kinase
CDT1	Chromatin licensing and DNA replication factor 1
CHD	Chromodomain helicase DNA-binding
CP	Cortical plate
Dvl	Dishevelled
FGF	Fibroblast growth factor
ID	Intellectual disability
IGF-I	Insulin-like growth factor-I
IP	Intermediate progenitor
IZ	Intermediate zone
LEF1	Lymphoid enhancer factor 1
LRP5	LDL receptor related protein 5
MAML	Mastermind like transcriptional coactivator
MAPK	Mitogen-activated protein kinase
MCM	Minichromosome maintenance
MCPH1	Microcephalin 1
mTOR	Mechanistic target of rapamycin
NDD	Neurodevelopmental disorder
NICD	Notch intracellular domain
NSC	Neural stem cell
NSPC	Neural stem progenitor cell
OLIG2	Oligodendrocyte transcription factor 2
PAX6	Paired box 6
PI3K	Phosphoinositol-3 kinase
PIP	Phosphatidylinositol phosphate
PKU	Phenylketonuria
PTCH	Patched
PTEN	Phosphatase and tensin homolog
RSPO	R-spondin
SETD5	SET domain containing 5
SHH	Sonic hedgehog
SMO	Smoothened
SVZ	Subventricular zone
SWI/SNF	Switch/Sucrose non-fermentable
TCF1	T cell factor
VPA	Valproic acid
VZ	Ventricular zone
WDR62	WD repeat domain 62
ZNRF3	Zinc and ring finger 3
